# Survey of attitudes, materials and methods employed in endodontic treatment by general dental practitioners in North Jordan

**DOI:** 10.1186/1472-6831-4-1

**Published:** 2004-09-10

**Authors:** Wael M Al-Omari

**Affiliations:** 1 Department of Restorative Dentistry, Faculty of Dentistry, Jordan University of Science and Technology, Irbid, Jordan

## Abstract

**Background:**

General dental practitioners provide the majority of endodontic treatment in Jordan. The aim of this study was to gather information on the methods, materials and attitudes employed in root canal treatment by dentists in North Jordan, in order to evaluate and improve the quality of current practice.

**Methods:**

A questionnaire was posted to all registered general dental practitioners working in private practice in Irbid Governate in North Jordan (n = 181). The questionnaire included information on methods, materials and techniques used in endodontic treatment.

**Results:**

Reply rate was 72% (n = 131). The results demonstrated that only five dentists used rubber dam occasionally and not routinely. The majority used cotton rolls for isolation solely or in combination with a high volume saliva ejector (n = 116). The most widely used irrigants were sodium hypochlorite and hydrogen peroxide, which were used by 32.9% (n = 43) and 33.6% (n = 44) of the respondents, respectively. Forty eight percent of the respondents (n = 61) used the cold lateral condensation technique for canal obturation, 31.3% (n = 41) used single cone, 9.9% (n = 13) used vertical condensation and 12.2% (n = 16) used paste or cement only for the obturation. The majority used zinc oxide eugenol as a sealer (72.5%). All, but one, respondents used hand instruments for canal preparation and the technique of choice was step back (52.7%). More than 50% (n = 70) of the dentists took one radiograph for determining the working length, whilst 22.9% (n = 30) did not take any radiograph at all. Most practitioners performed treatment in three visits for teeth with two or more root canals, and in two visits for teeth with a single root canal.

**Conclusions:**

This study indicates that dentists practicing in North Jordan do not comply with international quality standards and do not use recently introduced techniques. Many clinicians never take a radiograph for determining the working length and never used rubber dam or intra-canal medicaments.

## Background

Root canal treatment is considered an essential element in the dental services provided to the population in developed countries. Various investigations were, therefore, carried out to explore the standard of root canal treatment carried out by general dental practitioners in Europe [1-3].

It is the responsibility of the academics and dental schools to prepare their students to adopt the guidelines and recommended standards in root canal debridement, shaping and obturation [4,5]. Several studies have revealed that the majority of dentists do not comply with the formulated guidelines on the quality of root canal treatment [1-3,6]. These studies investigated the attitude of dentists in Western countries such as Germany [1], UK [2], Belgium [3] and the USA [6]. On the other hand, few studies have investigated the attitude of general dental practitioners toward various aspects of endodontic treatment in developing countries [7-9].

The majority of endodontic treatment in Jordan is provided by the general dental practitioners due to absence of specialists in endodontics and to the lack of postgraduate programs in Jordan. The purpose of the current study was, therefore, to investigate the attitude of dentists toward endodontic treatment and to explore the materials and methods employed by general dental practitioners in North Jordan and to compare these findings with well-acknowledged international academic standards.

## Methods

A postal survey of general dental practitioners in Jordan was carried out to investigate common materials and methods employed in root canal treatment. A questionnaire was developed and piloted by sending it to 20 general dental practitioners. According to the replies, the questionnaire was modified. Few questions were added and others were reworded. Additionally, the questionnaire was provided in Arabic and English language. The finally modified questionnaire was posted to all registered general dental practitioners (n = 181) listed in the records of the Jordanian Dental Association and working in private practices in Irbid Governate in North Jordan, 'Questionnaire [see Additional file 1]'. The questionnaire consisted of 28 questions concerning different aspects of endodontic treatment including the provision of molar endodontics, root canal therapy stages, materials, the choice of instruments, the use of rubber dam and isolation methods, number of appointments, number of radiographs taken throughout the treatment, the use of canal irrigants, the use of intracanal medicaments, the choice of obturation technique, temporary and permanent coronal restoration, and case monitoring and follow-up. There was a space made available in the questionnaire for free comments of respondents. The questionnaire was accompanied by an explanatory covering letter.

To investigate the influence of the years of practical experience on the materials and techniques employed, the sample was divided into groups based on the years of professional experience: group 1, up to 5 years; group 2, 6–10 years; group 3, 11–15 years; group 4, 16–20 years, and group 5, more than 20 years. The collected data were entered into a personal computer and analyzed using the statistical package SPSS. Simple descriptive statistics were used together with Chi-square (χ^2^) test. The chosen level of significance was set at *P *< 0.05. Unanswered questions were treated as missing values.

## Results

Of the 181 questionnaires distributed, 131 completed replies were received, which is a 72% response rate. The high response rate ensured that this study was representative for the general dental practitioners in North Jordan.

All the respondents performed endodontic treatment including molar teeth. However, none of the dentists reported that they would refer patients for a specialised endodontist opinion except cases, which were difficult or did not respond to initial treatment provided.

The distribution of the repondents according to the years of professional experience is shown in Table 1. Years in practice were not evenly distributed amongst the total respondents. The number of the first two groups (0–5 and 6–10) consisted of more than half the total respondents due to the significant increase in the number of graduates in the last 10 years. Seventy four percent of the respondents were males, 26% were females. These findings are consistent with the statistics obtained from the Jordanian Dental Association. In the current study, no statistically significant differences were found between the different periods of professional experience and any of the materials, instruments or techniques employed (*P *> 0.05).

**Table 1:** Data related to professional experience of the respondents.

***Years of Professional experience***	Frequency	Percentage %
0–5	43	32.8
6–10	28	21.4
11–15	26	19.8
16–20	20	15.3
>20	14	10.7

Table 2 shows the hand instruments used for preparation of the root canal. K-files were the most popular instruments. Root canal preparation was performed using K-files solely (30.5%) or in combination with other instruments (93.1%). Only one practitioner reported using engine-driven instruments (Profile, Dentsply Maillefer, ballaigues, Switzerland).

**Table 2:** The choice of root-canal preparation techniques and instruments

***Root canal preparation techniques***			***Root canal instrument***		
Technique	Frequency	%	Instrument	Frequency	%

Filing (push-pull)	36	27.5	File	40	30.5
Step back	69	52.7	Reamer	3	0.2
Step down	26	19.8	Hedström file	6	4.6
			File + hedström file	30	22.9
			File + reamer	20	15.3
			File, reamer + hedström file	32	24.4

The majority of dentists instrumented the canal using the step back technique. The next most popular preparation technique was the filing (push-pull) technique followed by the step down technique (Table 2).

The vast majority used gutta-percha points as their priniciple root filling material (87.8%), whilst 12.2% reported using only paste or cement to obturate the canal. Cold lateral compaction was the most common obturation technique (Table 3). The majority of dentists reported the use of a zinc oxide based sealer with the gutta-percha points (72.5%) followed by a calcium hydroxide based sealer, Sealapex (13.7%) (Table 3). Few dentists (n = 8) used the sealer Endomethasone as a paste root canal filling.

**Table 3:** The choice of obturation technique and type of sealer.

***Root canal obturation techniques***			***Type of sealer***		
Technique	Frequency	%	Type of material	Frequency	%

Single cone	41	31.3	Zinc oxide-eugenol	95	72.5
Lateral condensation	61	46.6	Sealapex	18	13.7
Vertical condensation	13	9.9	Endomethason	10	7.6
Cement only	16	12.2	Other	8	6.2

Intracanal medication was used by 63% of the respondents. The most common material used was tricresol formalin followed by calcium hydroxide. Other formulations were also used (Table 4).

**Table 4:** The frequency and percentages of intracanal medications used.

***Type of product***	Frequency	Percentage %
Calcium hydroxide	15	11.5
Formaldehyde	6	4.6
Tricresol formaline	45	34.4
Dexamethasone	1	0.8
Iodophorm	5	3.8
CMCP *	2	1.5
Other	8	6.1
None	49	37

* Camphorated monochlorophenol

Sodium hypochlorite and hydrogen peroxide solutions were used equally as an irrigating solutions. The most popular concentration of sodium hypochlorite was 3% which was used by 14.5% (n = 19) of the repondents, with only 2.3% (n = 3) using a 0.5% concentration. The most commonly used concentration of hydrogen peroxide was 3%, which was used by 21.4% (n = 28) of the respondents. The remainder used either normal saline or local anesthetic solutions (Table 5).

**Table 5:** Data related to the choice of root-canal irrigants.

Root-canal irrigants used			Concentration of NaOCl used			Concentration of H_2_O_2 _used		
Type	Frequency	%	Concentration (%)	Frequency	%	Concentration (%)	Frequency	%

Sodium hypochlorite	43	32.8	0.5	3	2.3	1	2	1.5
Normal saline	32	24.4	1	3	2.3	2	2	1.5
Hydrogen peroxide	44	33.6	2	8	6.1	3	28	21.4
Local anesthetic solution	2	1.5	3	19	14.5	4	2	1.5
None	10	7.6	5	3	2.3	6	10	7.6
			6	5	3.8	Do not use H_2_O_2_	87	66.4
			Do not use NaOCl	88	67.2			

**Figure 1:**
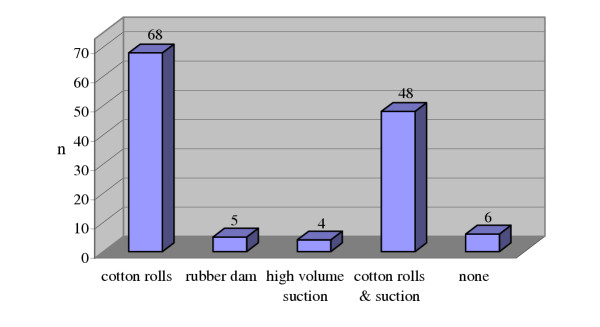
The number of dentists using different isolation methods.

None of the dentists reported using rubber dam routinely to isolate the field of operation during root canal therapy. However, only five dentists reported using rubber dam occasionally but not as a routine practice. The majority of the general dental practitioners used cotton rolls solely (n = 68) or cotton rolls in combination with a high volume saliva ejector (n = 116) to reduce contamination with saliva (Figure 1).

**Figure 2:**
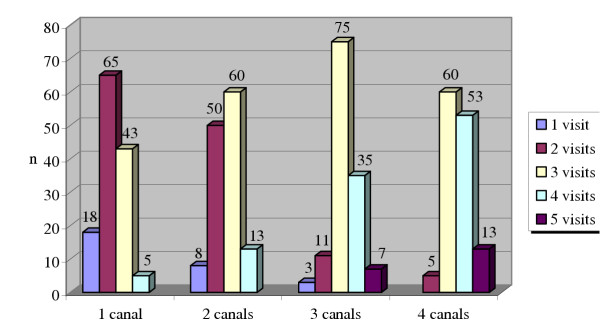
The number of visits according to the number of root canals per tooth.

The number of visits required to complete root canal treatment related to the number of root canals in a tooth is shown in Figure 2. It demonstrates that general dental practitioners complete root canal treatment in more than two visits for teeth with two or more root canals. However, half the respondents (49.7%) reported completing root canal treatment for teeth with single root canal in two visits.

Twenty seven percent of the practitioners took 3 radiographs for routine root canal treatment. 22.9% took only 2 radiographs. However, 23% reported taking only one preoperative radiograph with 4% taking only one radiograph for determining the working length. The remaining 22.9% of respondents undertook root canal treatment without taking any radiograph.

Only 14.5% of the respondents reported monitoring the root treated tooth radiographically after a period of 6 months. However, many of them mentioned that they would take a follow-up radiograph only if patients could afford to pay for it. The remainder indicated that they do not monitor their patients mostly for financial reasons and that patients would not return for follow-up appointment unless they have postoperative symptoms.

Zinc oxide eugenol cement was the most commonly placed temporary filling (92%). All dentists reported using amalgam for posterior teeth and composite for anterior teeth as a permanent coronal restorative material. All practitioners completed the restorations themselves. Sixty four percent of the respondents preferred to wait from 1 to 2 weeks after obturation before placing the permanent coronal filling, whilst the remainder placed the restoration immediately after completion of the treatment.

## Discussion

The response rate was 72%. It was higher than in many previous surveys conducted in Western countries with better communication infrastructure [2,7,10,11].

The vast majority of the respondents did not practice single visit root canal treatment. This finding was in agreement with the results of a previous study undertaken in another developing country, Sudan [9]. However, a study from the US [12] demonstrated a clear inclination to single visit endodontics, especially in cases without apical periodontitis. Single visit treatment appears to have gained more popularity and an increased credibility in the pre-clinical endodontic teaching in America and Europe [4]. Another survey [3], showed that a high percentage of Flemish dentists performed single visit root canal treatment. Multiple visit endodontic treatment could be a direct result of lacking adequate clinical time to complete the treatment in a single visit. The dentists may prefer to wait till the complete subsidence of pain and other symptoms before obturating the canal system. Another possible explanation could be that the initial visit was spent for treating the pain and acute symptoms [3].

Although the application of rubber dam is always recommended as a standard during root canal treatment procedure to provide isolation, protection and improve visual access, only five dentists reported using rubber dam very occasionally and not as a routine practice. Similar findings were found in Sudan (2%) and among Flemish dentists (3.4%) [6,8]. However, 59% of American dentists [6], 60% of dentists in UK [13] and 57% of general dental practitioners in New Zealand [14] reported using rubber dam routinely in endodontic treatment. The reasons for not using rubber dam could be the extra cost, additional time, lack of adequate skills or training, absence of patient's acceptability or inadequate education in the undergraduate teaching curriculum. It was found that continuing education course attendees seem to be encouraged to use rubber dam [14].

In the current survey, most general dental practitioners used hydrogen peroxide and sodium hypochlorite solutions as canal irrigants. The same result was demonstrated amongst dentists in Switzerland [11]. Sodium hypochlorite is recommended as the material of choice for irrigating the root canal system because of its effective antimicrobial and tissue solving action [15]. The selection of irrigant could be associated with the use of rubber dam, as it was found that 70% of rubber dam users among British dentists irrigated with sodium hypochlorite, whilst non-users tended to use local anesthetic solution [13]. The current findings do not mirror these findings. The vast majority of our respondents were non-users of rubber dam and one third of them use sodium hypochlorite routinely. A similar trend toward using sodium hypochlorite as an irrigant despite not using rubber dam for isolation, was noticed amongst Flemish dentists [16]. In the UK, the majority of dentists used local anesthetic solution to irrigate the canal space [2].

The use of either sodium hypochlorite or hydrogen peroxide without isolating the field of operation tightly with a rubber dam presents an obviously hazardous practice in the use of potentially irritant irrigation solutions.

Despite the fact that calcium hydroxide is recognized as the standard intracanal medicament for inter-appointment dressing [17], it was used by only 11.5% of the respondents. More than one third of the general practitioners reported using formaldehyde-containing materials. This finding is consistent with previous findings recorded for Sudanese dentists [9]. Although formaldehyde-containing products have been used for their antimicrobial and fixative properties, they are toxic to periradicular tissues [18] and may have mutagenic and carcinogenic potential [19]. The use of calcium hydroxide, as intracanal medication, should be encouraged among dentists in developing countries such as Jordan, as it is effective against most root canal pathogens and able to denature bacterial endotoxins [20,21]. It has, also, been reported to be the material of choice by dentists in the Western world [11,22].

The step back technique was the most popular canal preparation technique among North Jordanian general dental practitioners. The filing (push-pull) technique, on the other hand, was used by 27.5% of the respondents. In another study, 60.4% of Flemish dentists used the standard filing technique [16]. Generally, dentists in Jordan tended to use hand instruments and were not inclined to use more advanced engine driven techniques for shaping the root canal system.

Almost half of the general dental practioners in North Jordan used cold lateral compaction of gutta-percha to obturate the root canal space. This technique is acknowledged universally and is the most common obturation technique [4]. However, 31.3% of the dentists in the current survey used a single cone technique, in common with 68% of Swiss dentists [11]. Additionally, 12.2% of respondents used only paste to obturate the root canal system. Seemingly, dentists in North Jordan are not strong advocates of the more recently introduced advanced obturation techniques. This may be attributed to additional cost involved or the lack of skill and training.

## Conclusions

This study investigated the status of endodontic practice among general dental practitioners working in private offices in North Jordan. It demonstrated that dentists performed procedures which often deviated from well-acknowledged endodontic quality guidelines. Dentists did not use rubber dam for isolation and frequently use formaldehyde-containing materials for inter-appointments dressing. In addition, a significant proportion of dentists (n = 30) did not use radiographs at any stage of endodontic treatment. General practitioners did not seem to keep up with recently introduced techniques, but use more conventional methods.

The North Jordanian general dental practitioners carried out endodontic treatment with few referals to specialists. However, the absence of postgraduate endodontic programs and continuing education courses in addition to economic restrictions could explain why dentists in Jordan do not carry out endodontic treatment in accordance with recognized international standards.

## Competing Interests

None declared.

## Pre-publication history

The pre-publication history for this paper can be accessed here:



## Supplementary Material

Additional File 1 Endodontic Survey Questionnaire text of the file contains questions related to endodontic practice among general dental practitioners. Click here for file

## References

[B1] Weiger R, Hitzler S, Hermle G, Löst C (1997). Periapical status, quality of root canal fillings and estimated endodontic treatment needs in an urban German population. Endod Dent Traumatol.

[B2] Jenkins SM, Hayes SJ, Dummer PM (2001). A study of endodontic treatment carried out in dental practice within the UK. Int Endod J.

[B3] Slaus G, Bottengerg P (2002). A survey of endodontic practice amongst Flemish dentists. Int Endod J.

[B4] Qualtrough AJ, Whitworih JM, Dummer PM (1999). Preclinical endodontology: an international comparison. Int Endod J.

[B5] European Society of Endodontology (1994). Consensus report of the European Society of Endodonology on quality guidelines for endodontic treatment. Int Endod J.

[B6] Whitten BH, Gardiner DL, Jeansonne BG, Lemon RR (1996). Current trends in endodontic treatment: report of a national survey. J Am Dent Assoc.

[B7] Akpata ES (1984). Endodontic treatment in Nigeria. Int Endod J.

[B8] Maina SW, Ng'ang'a PM (1991). Root canal treatment and pulpotomy in Kenya. East Afr Med J.

[B9] Ahmed MF, Elseed Al, lbrahim YE (2000). Root canal treatment in general practice in Sudan. Int Endod J.

[B10] Pitt Ford TR, Stock CJ, Loxley HC, Watsson RM (1983). A survey of endodontics in general practice in England. Br Dent J.

[B11] Barbakow F (1996). The status of root canal therapy in Switzerland in 1993. J Dent Assoc S Afr.

[B12] Gatewood RS, Himel VT, Dorn SO (1990). Treatment of the endodontic emergency: a decade later. J Endod.

[B13] Whitworth JM, Seccombe GV, Shoker K, Steele JG (2000). Use of rubber dam and irrigant selection in UK general dental practice. Int Endod J.

[B14] Koshy S, Chandler NP (2002). Use of rubber dam and its association with other endodontic procedures in New Zealand. N Z Dent J.

[B15] Byström A, Sundqvist G (1983). Bacteriologic evaluation of the effect of 0.5 percent sodium hypochlorite in endodontic therapy. Oral Surg Oral Med Oral Path.

[B16] Hommez GM, Braem M, De Moor RI (2003). Root canal treatment performed by Flemish dentists. Part I. Cleaning and shaping. Int Endod J.

[B17] Chong BS, Pitt Ford TR (1992). The role of intracanal medication in root canal treatment. Int Endod J.

[B18] Gulabivala K (1995). lntracanal medication and temporary seal. In Color Atlas and Text of Endodontics.

[B19] Spangberg I, Malvern PA (1994). Intracanal medication. In Endodontics.

[B20] Bystrom A, Claesson R, Sundqvist G (1985). The antibacterial effect of camphorated paramonochlorphenol, camphorated phenol and calcium hydroxide in the treatment of infected root canals. Endod Dent Traumatol.

[B21] Sjögren U, Figdor D, Spangberg L, Sundqvist G (1991). The antimicrobial effect of calcium hydroxide as a short-term intracanal dressing. Int Endod J.

[B22] Hommez GM, Braem M, De Moor RJ (2003). Root canal treatment performed by Flemish dentists. Part 2. Canal filling and decision for referrals and treatment of apical periodontitis. Int Endod J.

