# Supplementary material for: Survey of attitudes, materials and methods employed in endodontic treatment by general dental practitioners in North Jordan
Source: BMC Oral Health. 2004 Sep 10;4:1. doi: 10.1186/1472-6831-4-1 (PMC518977; doi:10.1186/1472-6831-4-1)
Supplement: Additional File 1 — Endodontic Survey Questionnaire text of the file contains questions related to endodontic practice among general dental practitioners. [file 1472-6831-4-1-S1.doc]

# English translation from the Arabic questionnaire

# Endodontic Treatment Status Survey

# Department of Restorative Dentistry

# Jordan University of Science and Technology

*Dear participant,*

This questionnaire is for collecting information about the materials and methods employed in endodontic treatment by general dental practitioners in North Jordan. Your name and address are optional information, though they could be of help if further detailed information is to be collected later. All information will be confidential. Please, answer by ticking one or more alternatives whenever appropriate and/or by writing your answer on the line below question.

Name (Optional)______________________Qualifications______________________

Years of professional experience_____________________Gender________________

Address (Optional)_____________________________________________________

Q1. Do you perform endodontic treatment for your patients?

- Yes
- No

Q2. If the answer to Q1 is No, what are the reasons for not doing endodndontic treatment?

- Lack of time
- Lack of adequate skills and training
- Inadequate knowledge
- Others (specify)__________________________________________________

Q3. Do you undertake molar endodnontic treatment?

- Yes
- No

Q4. What are the methods of isolation do you use?

- None
- Rubber dam
- Cotton rolls only
- High volume suction only
- Cotton rolls and high volume suction
- Other (specify)__________________________________________________

Q5. If the method of isolation used is rubber dam, how often do you use it?

- Sometimes (occasionally)
- Always (for all patients)
- Never

Q6. What is the technique used for preparation of the root canals?

- Filing (push-pull)
- Step-back
- Step-down
- Other (specify)_________________________________________________

Q7. What type of instruments do you use?

- Hand instruments only
- Engine-driven (Rotary) instruments________________________________
- Both hand and rotary instruments

Q8. What root canal hand instrument do you use?

- K-File
- Reamer
- Hedström file
- File and hedström file
- Reamer and hedström file
- Reamer and file
- Reamer, file and hedström file

Q9.At what stage of the root canal treatment do you take a radiograph?

- Preoperative
- Working length determination
- Master cone determination
- Postoperative (after obturation)
- None

Q10. What inracanal irrigant do you use?

- Normal saline
- Hydrogen peroxide
- Sodium hypochlorite
- Chloramine
- Chlorehexidine
- Local anesthetic solution
- Combination (specify)_____________________________________________
- None
- Other (specify)___________________________________________________

Q11. If sodium hypochlorite is the irrigant of choice, what is the concentration used?

________________________________________________________________

Q12. If hydrogen peroxide is the irrigant of choice, what is the concentration used?

________________________________________________________________

Q13. What inracanal medication do you use?

- Calcium hydroxide (Ca (OH)_2_)
- Dexamethason
- Formaldehyde
- Tricresol formaline
- Iodpphore
- Camphorated monochlorophenol (CMCP)
- None
- Other (specify)___________________________________________________

Q14. What is the root canal obturation material used?

- Gutta-percha
- cement only
- Silver points
- Other (specify)___________________________________________________

Q15. What is the gutta-percha obturation technique do you use?

- Single-cone
- Lateral condensation
- Vertical condensation
- Thermomechanical compaction
- Thermafill
- Other (specify)___________________________________________________

Q16. What is the type of sealer used with gutta percha points?

- Zinc-oxide eugenol
- Endomethason
- Topseal
- Sealapex
- Other (specify)___________________________________________________

Q17. What is the temporary filling used to seal the coronal access cavity between

appointments?

- Zinc-oxide eugenol
- Intermediate restorative material (IRM)
- Glass Ionomer
- Cavit
- Other (specify)___________________________________________________

Q18. How many appointments is needed to complete root canal treatment for a

single-rooted teeth?

___________________________________________________________________

Q19. How many appointments is needed to complete root canal treatment for teeth

with two root canals?

____________________________________________________________________

Q20. How many appointments is needed to complete root canal treatment for teeth

with three root canals?

____________________________________________________________________

Q21. How many appointments is needed to complete root canal treatment for teeth

with four or more root canals?

____________________________________________________________________

Q22. Do you follow-up the root treated tooth, and monitor it radiographically?

- Yes
- No

Q23. If answer to Q23 is yes, the patient is followed up radiographically every:

- 1 month
- 2 months
- 4 months
- 6 months
- 1 year
- other _____________________________________________________

Q24. If the answer to Q23 is no, give reasons?

- Lack of time
- Unmotivated patients
- Cost of the radiograph
- Other______________________________________________________

Q25. Do you restore the coronal access cavity yourself?

- Yes
- No

Q26. Do you restore the coronal access cavity immediately after completion of RCT?

- Yes
- No

If answer is No, how long do you wait after obturation, to restore the coronal access cavity? _____________________________________________________________

Q27. What is the coronal restoration of choice?

1. Anterior teeth________________________________________________
2. Posterior teeth________________________________________________

Q 28. When do you refer a patient t a specialist?

- Very difficult cases
- Cases that do not respond to initial treatment
- Unmotivated patients
- Other (specify)__________________________________________________

Any additional comments**……………………………………………………………..**

**…………………………………………………………………………………………**

Note: Please, do not hesitate to contact me if you have any enquiries about the questionnaire.

Cordially Yours

# Dr Wael Al-Omari

***Telephone:0795521461***

***Email:womari@just.edu.jo***
